# Intensive inpatient treatment improves emotion-regulation capacities among adults with severe mental illness

**DOI:** 10.1186/2051-6673-1-19

**Published:** 2014-12-15

**Authors:** J Christopher Fowler, Jon G Allen, John M Hart, Hanna Szlykh, Thomas E Ellis, B Christopher Frueh, John M Oldham

**Affiliations:** The Menninger Clinic, 12301 Main Street, Houston, TX 77035 USA; Baylor College of Medicine, One Baylor Plaza, Houston, TX 77030 USA; Department of Psychology, University of Hawaii, 200 West Kawili St, Hilo, HI 96720 USA

**Keywords:** Attachment Insecurity, Experiential Avoidance, Emotion Regulation, Treatment Response

## Abstract

**Background:**

Impaired capacity for emotion regulation is associated with a broad spectrum of psychiatric disturbances; however, little is known about treatment response in emotion regulation functioning among patients with severe mental illness. This study examined treatment response and the role that experiential avoidance plays in mediating the relationship between attachment anxiety/avoidance and change in emotion regulation.

**Methods:**

Difficulties in emotion regulation were assessed at admission and at discharge, and rates of improvement and deterioration in emotion regulation were calculated. Attachment anxiety and avoidance were assessed in conjunction with experiential avoidance at baseline in a large cohort (N = 493) of adults admitted to a specialized adult psychiatric hospital.

**Results:**

Inpatient treatment was associated with clinically significant improvement in emotion-regulation capacities for 49 percent of patients completing at least four weeks of treatment. Fifty-six percent of patients attained a status of recovery. Greater attachment avoidance and anxiety were related to positive change in emotion regulation at discharge. Experiential avoidance fully mediated the relationship between insecure attachment and change in emotion-regulation capacities.

**Conclusions:**

Contrary to expectation, greater attachment insecurity (anxiety and avoidance) as well as greater experiential avoidance predicted improvement in emotion regulation. These counterintuitive findings add to a growing evidence base indicating that severity of psychopathology is associated with greater improvement in hospitalized patients. Results of the mediation analysis suggest that targeting experiential avoidance may be an effective augmentation in the treatment of impaired emotion regulation functioning.

## Background

Among psychiatric disorders, borderline personality disorder (BPD) is a prototype for conspicuous impairment of emotion regulation. For example, all seven pathological personality traits of BPD specified in the DSM-5 alternative model for personality disorders [1] are potentially associated with impaired emotion regulation: four relate to negative affectivity (i.e., emotional lability, anxiousness, separation insecurity, and depressivity), two relate to disinhibition (impulsivity and risk taking), and one relates to antagonism (hostility). In clinical work, impaired emotion regulation has for some time been an important target for interventions in the treatment of BPD [2–8]. Yet impaired emotion regulation is by no means confined to BPD but rather is associated with other disorders such as substance use [9, 10], generalized anxiety disorder [11], posttraumatic stress disorder [12, 13], and, more generally, with a wide range of symptomatology that cuts across psychiatric disorders [14–17].

Accordingly, improving emotion-regulation capacities is an important transdiagnostic treatment outcome in its own right [18–24]. Nonetheless, only a small proportion of efficacy and effectiveness studies assess change in emotion regulation as a primary outcome. Although the results are promising, the samples tend to be small, exclude patients with more than two psychiatric disorders, and in some cases, eliminate patients with suicide risk. Hence relatively little is known about the effectiveness of psychiatric interventions on emotion regulation among patients with severe mental illness (SMI). Kessler [25] defined SMI as meeting one or more current DSM-IV/CIDI mental disorders in combination with one or more of the following criteria within the last 12 months: suicide attempt with serious lethality of intent; work disability or substantial limitation as the result of a mental disorder, bipolar I disorder, a behavioral disorder with associated serious violence or criminal behavior; or any disorder that resulted in 30 days out of role in the year. Following these criteria, the current study investigated improvement in emotion regulation as the primary outcome in a large, diagnostically heterogeneous and co-morbid sample of psychiatric in patients with SMI.

Gross [26] defined emotion regulation as “the activation of a goal to up- or down-regulate either the magnitude or duration of the emotional response” (p. 359), and he elucidated the sheer complexity of the processes involved in modifying the trajectory of emotions [26, 27]. Gratz and Roemer [28] operationalized the multifaceted impairment of emotion regulation in the Difficulties in Emotion Regulation Scale (DERS), which includes six components: nonacceptance of emotional responses, difficulties engaging in goal-directed behavior, impulse control difficulties, lack of emotional awareness, limited access to emotion-regulation strategies, and lack of emotional clarity. Deficits in one or more of these capacities contribute to impaired emotion regulation. Adaptive emotion regulation includes awareness, understanding, and acceptance of emotions as contrasted with over-control, avoidance, and harsh judgment of emotions [28, 29].

Therapeutic efforts and interventions that focus on exploration, understanding, acceptance, and modulation of intense emotions cut across multiple forms of psychotherapy for BPD [4] and are core features of two unified protocols for psychotherapy of broad-based treatment for transdiagnostic psychiatric disorders [19, 20]. In our view, awareness and acceptance of internal experience is a foundation of deliberate and adaptive efforts to regulate emotion. Burgeoning theory and research on experiential acceptance versus avoidance, highlighted in Acceptance and Commitment Therapy [30] bear directly on emotion dysregulation and associated psychopathology. Hayes and colleagues defined experiential avoidance (EA) as the attempt to control the form or frequency of aversive private experiences (e.g., bodily sensations, emotions, thoughts, memories, and behavioral predispositions), despite the cost of interfering with actions associated with valued activities and goals. Conversely, experiential acceptance is defined as the willingness to experience unwanted thoughts and feelings in order to pursue valued goals. Hence experiential acceptance and avoidance as measured by the Acceptance and Action Questionnaire [31] are construed as exemplifying psychological flexibility and inflexibility, respectively. As is true of emotion dysregulation more broadly, EA is associated with a wide range of psychopathology [32–38], whereas experiential acceptance is associated with adaptive functioning, such as pro-social behavior and sense of wellbeing [39].

Although *self*-regulation of emotion has been a major focus of research and treatment, Gross [26] proposed that *interpersonal* regulation of emotion also deserves serious consideration. In this vein, Coan and Maresh [40] contended that “Attachment theory provides the quintessential example of socially regulated emotion in its description of mother-child attachment interactions, in which infants seek attachment figures during periods of distress and are soothed by their caregiver’s presence” (p. 226). Accordingly, extensive literature points to attachment relationships as playing a key role in emotion regulation, not only in infancy and childhood but also through adulthood [41–47]. Moreover, recent research suggests that attachment security is conducive to greater emotion-regulation capacity insofar as security is conducive to awareness and understanding of emotional distress. That is, early research identified the caregiver’s sensitive responsiveness to the infant’s distress as a critical contributor to the infant’s attachment security [47]. More recently, Fonagy and colleagues pinpointed the caregiver’s mentalizing activity—awareness and understanding of mental states in self and others—as the aspect of sensitivity that is most central to the development of security [48–50]. Subsequent research has shown that parental mentalizing of the child is conducive to the child’s attachment security [51–53]; moreover, parental mentalizing and child attachment security are conducive to the child’s developing mentalizing capacity [53]. This capacity includes “mentalized affectivity” —mentalizing in the midst of the emotional state—which overlaps with experiential acceptance [54].

Yet the effectiveness of attachment in promoting awareness and regulation of emotion hinges on the security of the attachment relationship. Along with secure attachment, two patterns of insecure attachment have been identified [47]: avoidant infants deactivate their attachment responses, do not rely on the caregiver for security and comforting, and strive to be self-reliant; anxious-ambivalent infants hyperactivate their attachment responses, cling to the caregiver but resist soothing, and fail to develop self-reliance. Although there are complex developmental trajectories with an intermingling of continuity and lawful discontinuity from infancy and childhood to adulthood [55, 56], analogous patterns of secure and insecure attachment have been identified in adulthood [46], assessed by structured clinical interview [57] and a range of self-report measures [58–60]. Paralleling childhood, avoidant attachment in adulthood is associated with suppression of emotion, whereas anxious attachment is associated with heightened and exaggerated emotional reactivity [46]. Relatedly, a small but growing body of research highlights the negative impact that attachment insecurity can play in adult treatment outcomes [61, 62]; therefore, it is important to examine the relationship between attachment insecurity and various outcomes including the impact on emotion-regulation capacities. While the body of empirical results attests to the relationships between attachment and emotion regulation, a significant knowledge gap exists in that much remains to be discovered about processes that mediate the relationships between attachment insecurity and positive changes in emotion-regulation capacities. Given the literature connecting EA and emotion regulation, we chose EA as a logical first step in exploring mediation of attachment insecurity and emotion regulation.

In linking attachment and experiential acceptance with emotion regulation, the present study has two overall aims. First, we hypothesized that intensive, non-acute, voluntary inpatient treatment would improve emotion-regulation capacities in a sample of patients with SMI. This hypothesis is based on the psychotherapeutic nature of the hospital treatment, which not only aims to foster self-regulation but also capitalizes on group interventions in the context of a therapeutic milieu. Insofar as the treatment as a whole promotes social engagement, it fosters interpersonal regulation of emotion. Second, the study investigates the relationships among attachment insecurity, experiential avoidance, and improvement in emotion regulation. While experiential avoidance and emotion regulation share some conceptual overlap, the literature and research clearly indicate that other psychological and interpersonal functions play a critical role in emotion regulation; therefore, we tested the hypothesis that EA mediates the relationship between insecure attachment and change in emotional regulation inasmuch as the limited awareness, understanding, and acceptance of emotional distress associated with either form of insecure attachment would impinge on efforts to develop more effective emotion-regulation strategies in treatment relationships. Due to significant co-morbidity within the population (see Results), separate analyses for specific clinical disorders were eschewed in favor of a focus on the underlying cross-cutting dimension of SMI.

## Method

### Participants

Participants were 493 individuals admitted to a specialized psychiatric hospital (June 2012-June 2013) with length of stay of 28 days or greater. Average length of stay for the sample was 53 days (SD = 16.9). Gender distribution was relatively even: 255 were women (52%) and 238 were men (48%). Average age was 29.2 years (*SD* = 13.1). Participants were Caucasian (*n* = 449, 91%), multiracial (*n* = 30, 6%), African American (*n* = 7, 1.4%), Asian (*n* = 5, 1%), American Indian (n = 1, .2%) and Hawaiian or other Pacific Islander (n = 1, .2%). Five percent of participants identified as being of Hispanic or Latino ethnicity. A majority (64%) of participants were not working prior to admission.

### Treatment setting and procedures

Typical lengths of stay in the hospital range from four to eight weeks. Treatment included medication management, individual and group psychotherapy, psychoeducation, and social activities in the context of a therapeutic milieu that promotes expression and understanding of emotional reactions. Psychoeducational groups on mentalizing [63, 64] and skills drawn from Dialectical Behavior Therapy [56] and Mentalization-Based Therapy [65, 66] directly address impairments in emotion regulation.

Data were collected as part of the hospital’s Adult Outcomes Project, described in detail elsewhere [67]. All participants were assessed using validated measures within 72 hours of admission, followed by re-administration of selected measures at 14-day intervals during treatment and at point of discharge. This project was a clinical outcomes project, conducted with all patients. Use of the project’s data was approved by Baylor College of Medicine’s Institutional Review Board.

### Measures

#### Difficulties in Emotion Regulation Scale (DERS)

The DERS is a 36-item self-report measure assessing difficulties in emotion regulation, demonstrating good internal consistency (Cronbach’s *α* from.80 to .89), test-retest reliability (*r* = .88), and construct validity [28]. Factor analytic studies [28, 68–70] support a factor structure consisting of six dimensions: 1. Nonacceptance of emotional responses, 2. Difficulty engaging in goal-directed behavior when experiencing negative emotions, 3. Impulse control difficulties when experiencing negative emotions, 4. Lack of emotional awareness, 5. Limited access to emotion regulation strategies, and 6. Lack of emotional clarity. Items are rated on a 5-point Likert scale, ranging from 1 (almost never, 0-10%) to 5 (almost always, 91-100%). The scale yields a total score (range 36–180) with higher scores indicative of the degree of impairment in emotion regulation. Scores falling between 75–80 are indicative of a healthy range of functioning [28].

#### Relationship Questionnaire (RQ)

The RQ [58] is a prototype measure derived by crossing two theoretical dimensions of attachment representations: attachment anxiety (positive/negative) and attachment avoidance (positive/negative). The questionnaire was administered at admission. Respondents rate each prototype (secure, dismissing, preoccupied, and fearful) on a 7-point scale regarding the extent to which each description corresponds to their general relationship style. Total scores are derived by the following formulas: Attachment Anxiety = (Secure + Dismissing) - (Preoccupied + Fearful); Attachment Avoidance = (Secure + Preoccupied) - (Dismissing + Fearful). Scores on each dimension of the RQ range from -12 to +12. Negative attachment anxiety scores are associated with attachment-related anxiety based on doubts that the self is worthy of attention and affection, creating worries that relationship partners will not be available in times of need. Negative attachment avoidance scores are associated with attachment-related avoidance and are rooted in a person’s distrust of relationship partners’ goodwill, which causes him or her to maintain behavioral and emotional independence and distance from others.

#### Acceptance and Action Questionnaire-II (AAQ-II)

The AAQ-II [31] is a 7-item self-report measure of experiential acceptance versus avoidance. The AAQ-II has demonstrated good internal consistency (Cronbach’s α = .84) across six samples and high test-retest reliability coefficients across three months (r = .81) and six months (r = .79). Higher scores are associated with greater experiential avoidance, which refers to attempts to alter, control and suppress difficult private events, such as thoughts, feelings, and sensations.

#### Structured Clinical Interview for DSM-IV Axis I and Axis II Disorders (SCID-I and SCID-II)

The SCID-I [71] and SCID II [72] interviews were conducted by master’s level researchers after reviewing pertinent psychiatric and psychosocial evaluations. This process combined the ecologically valid longitudinal evaluation of all available data diagnostic approach [73] with the rigorous diagnostic interviews of SCID I and SCID II.

### Data analysis

Analyses were conducted using SPSS for windows, version 21 (IBM). Paired T-Tests were used to assess average change in DERS scores and to estimate effect-size change between admission and discharge. Average pre-post change obscures patient-level rates of change. In addition, presenting raw pre-post change can be somewhat misleading and unreliable due to measurement error in the form of poor test-retest reliability and sample artifacts such as regression to the mean in highly symptomatic patient samples. To address these potential shortcomings, reliable change index scores (RCI) and remission rates were calculated for each patient. Briefly, RCI relates to individual patient functioning that is statistically reliable such that change between pre-treatment and post-treatment scores reflects true change rather than an artifact of measurement error. While there are several formulas for computing RCI, the Edwards-Nunnally formula [74] is a conservative method that corrects for regression toward the mean. The Edwards-Nunnally RCI formula requires the following computations: 1. Adjusting for regression to the mean by computing adjusted pre-treatment mean (X_adjpre=_ Test-Retest Reliability * [Individual’s score – Mean of Group] + Mean of Group), 2. Standard error of measurement (SE = SD √1 – Test-Retest Reliability), 3. Standard error of the difference between the two test scores (S_diff_ = √2 [SE^2^]), 4. Reliable Change Index (RCI = X_post_ – X_adjpre_/ S_diff_) where X_adjpre_ = the adjusted pretest score, X_post_ = the posttest score, S_diff_ = the standard error of the difference between the two test scores. Test-retest reliability^28^ (r = .88) was used in all calculations for RCI. An RCI score greater than 1.96 indicates statistically reliable change. We also calculated clinical improvement defined as an RCI score >1.28 [75]. Normative functioning was indicated when patient functioning returned to the normative range—Nonclinical samples of college students and community adults have scores averaging between 75–80 on the DERS [28].

Mediation analyses were carried out using AMOS structural equation modeling with bootstrap sampling (IBM). A simple path model was constructed to determine if attachment anxiety and avoidance predict change in emotion regulation (DERS RCI). A second path model tested the hypothesis that experiential avoidance mediates the relationship between attachment anxiety, attachment avoidance, and change in DERS RCI. Mediation is indicated when the independent variable is significantly related to the mediator, the independent variable is significantly related to the dependent variable, and the effect of the independent variable on the dependent variable is weakened when the proposed mediator is controlled [76]. The magnitude of mediation was assessed by examining the direct and indirect effects of the path analysis [77]. The conventional rule-of-thumb guidelines suggest that a fit is acceptable if CFI is .90 or greater, and RMSEA is .10 or less [78].

## Results

Diagnostic profiles and past psychiatric history (Table 1) indicated high levels of functional impairment and co-morbidity consistent with severe mental illness [25]. Eighty-eight percent of patients in the sample were diagnosed with at least two co-occurring Axis I disorders with average of 3.6 (SD = 2.2). Six percent manifested a psychotic spectrum disorder, 19% with a bipolar spectrum disorder, 57% with a substance use disorder, 61% with an anxiety spectrum disorder, and 65% with a major depressive disorder. Personality disorders were present in 34 percent of the sample, including borderline (20%), avoidant (14%), obsessive-compulsive (6%), narcissistic (4%), antisocial (3%), personality disorders not otherwise specified (1%), and schizotypal (.7%). Other markers indicative of severe mental illness included a high number of previous psychiatric hospitalizations (M = 1.2, SD = 2.9) and high rates of active suicidal ideation (63%). On average, patients reported high levels of attachment anxiety (M = -1.1, SD = 4.7) as well as attachment avoidance (M = -.41, SD = 4.8). Computation of the attachment status indicated that 79% of patients were categorized as insecurely attached (attachment anxiety and/or attachment avoidance scores in the negative range).

**Table 1:** **Descriptive Statistics (N = 496)**

Variable	Minimum	Maximum	Mean	SD
Length of Stay	28	166	53.2	16.9
Age	19	59	35.8	14.0
Total DSM-IV Axis I/II	0	13	3.6	2.2
Number of Hospitalizations	0	50	1.2	2.9
Attachment Anxiety	-10	12	-.1.1	4.7
Attachment Avoidance	-11	12	-.41	4.8
AAQ-2	7	49	32.0	10.3
Admission DERS	36	171	103.0	26.9
Discharge DERS	36	152	75.4	24.9
DERS RCI*	-4.1	7.7	2.06	1.80

*DERS Reliable Change Index is compute as a Z score.

Admission and discharge scores on the DERS are displayed in Table 1. Assessment of change from admission to discharge on DERS total score across all patients produced statistically significant improvement (t = 23.9, p < .0001). Effect-size change was large (d = 1.07), indicating an average of 1 standard deviation reduction in impaired emotion regulation. Improvement and deterioration rates for the DERS change during the course of treatment appear in Table 2. Conservative estimates of reliable change in DERS (RCI >1.96) demonstrated that 241 patients (48.9%) met this criterion for improvement, whereas 2 patients deteriorated. Clinically significant improvement as evidenced by attainment of normative range of functioning (DERS <76) indicated that 275 patients (55.8%) met this criterion, whereas seven patients shifted from normative to a pathological level of functioning. Alternate classification (RCI >1.28) showed that 317 patients (64.3%) demonstrated positive improvement in emotion regulation at the point of discharge, and 303 (61.5%) had DERS total scores below 81. By this criterion, a total of 7 patients demonstrated deterioration in their capacity to regulate emotion between admission and point of discharge.

**Table 2:** **Rates of Change in Difficulty in Emotion Regulation Scale (N = 493)**

	DERS		DERS	
	Improved	(%)	Deteriorated	(%)
RCI >1.96	241	48.9	2.0	0.004
DERS <76	275	55.8	7	0.01
RCI >1.28	317	64.3	4.0	0.008
DERS <81	303	61.5	7	0.01

Note: Difficulty in Emotion Regulation Scale (DERS); Reliable Change Index (RCI);

Deterioration rates are based on RCI > -1.96, and movement from normative range at baseline to pathological range (>75, 80) at discharge.

**Figure 1:**
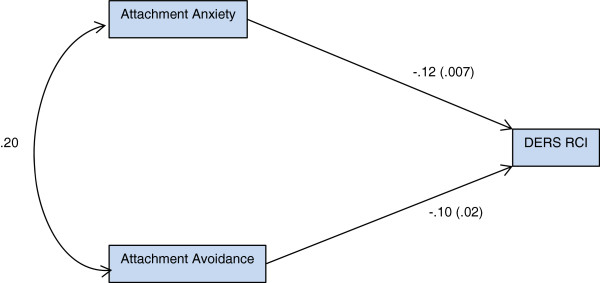
**Path diagram of attachment predicting change in emotion regulation.**

The simple path model (Figure 1) demonstrated that greater attachment anxiety at admission is predictive of greater change in emotion regulation at point of discharge (β = -.12, p = .007). Greater attachment avoidance at admission also was predictive of greater change in emotion regulation at the point of discharge (β = -.11, p = .02). Model fit indices resulted in reasonable fit (model had 0 degrees of freedom therefore *χ*^*2*^ could not be computed), CFI =1.0, and RMSEA = .068 (90% C.I. =.042-.095).

**Figure 2:**
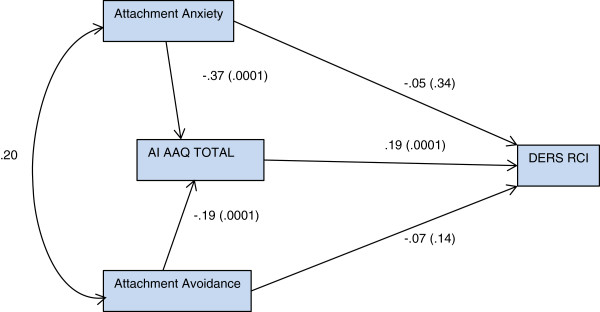
**Path diagram of ea mediating attachment relationship to emotion regulation change.**

The second path model (Figure 2) testing for potential mediation revealed that attachment anxiety was significantly related to experiential avoidance (β = -.37, SE = .086, p = .0001), attachment avoidance was significantly related to experiential avoidance (β = -.19, SE = .084, p = .0001), and greater experiential avoidance at admission was highly predictive of greater improvement in emotion regulation at discharge (β = .19, SE = .009, p = .0001). In this model, attachment anxiety was not significantly related to change in emotion regulation (β = -.05, SE = .018, p = .34) when experiential avoidance was included in the path. Similarly, attachment avoidance was not significantly related to change in emotion regulation (β = -.07, SE = .017, p = .14) when experiential avoidance was included. Examination of the indirect effects indicated that experiential avoidance fully mediated the effect of attachment anxiety on change in emotion regulation (p < 0.001), that is, the relationship between attachment anxiety and change in emotion regulation was nullified by the inclusion of experiential avoidance. Similarly, experiential avoidance fully mediated the effect of attachment avoidance on change in emotion regulation (p < 0.0001). Model fit indices for the mediation model resulted in a questionable fit, (model had 0 degrees of freedom therefore *χ*^*2*^ could not be computed), CFI =1.0, and RMSEA = .22 (90% C.I. = .197-.258).

Given the relative poor fit model 2, post-hoc analysis of a third mediation model utilizing three control variables (gender, length of hospitalization in days, and number of psychiatric disorders) tested impact on change in emotion-regulation functioning. These potential covariates were selected based on literature indicating gender differences in emotion regulation functioning [79], that treatment dose is associated with outcome [80] and severity of psychiatric disturbance is a strong predictor of treatment outcome [81]. The path model was not significantly different from Model 2 because the control variables were not significantly associated with change in emotion regulation (gender: β = -.21, SE = .077, p = .23; length of hospitalization: β = .001, SE = .004, p = .83; number of psychiatric disorders: β = .002, SE = .043, p = .96). Model fit indices resulted in slightly better fit than model 2; however it still resulted in questionable fit (CFI =1.0, and RMSEA = .22 [90% C.I. = .197-.258]).

## Discussion

This large-scale open trial demonstrated significant treatment response in emotion-regulation functioning among adult psychiatric in patients with severe mental illness. On average, patients experienced a 1 standard-deviation improvement in emotion regulation at the point of discharge. Forty-nine percent of all patients evidenced a clinically significant improvement in emotion regulation, with approximately 56% of all patients attaining scores in the healthy range of functioning at discharge. Clinical and statistical change appears to reflect true change rather than statistical artifacts because the authors utilized the Edward-Nunnally formula that controls for regression to the mean. The study yielded unexpected findings insofar as higher levels of impaired functioning that contribute to problems in emotion regulation were associated with greater change in emotion regulation capacities. That is, both forms of attachment insecurity as well as higher levels of experiential avoidance were associated with greater improvement in emotion regulation. Similar counterintuitive findings were reported in open trials in inpatient settings [82–84]. Furthermore, a large scale meta-analysis [85] of randomized control trials for the treatment of depression revealed that post-treatment effect sizes were larger for high-severity patients, leading the authors to conclude, “Contrary to conventional wisdom, our findings suggest that when compared with control conditions, psychological treatment might be more efficacious for high-severity than for low-severity patients.”

Taking individual differences into account, what might account for such dramatic improvement in emotion-regulation capacities among patients with severe mental illness and treatment-resistant disorders? A plausible explanation is that the treatment exerted a mutative effect for patients who struggled with greater attachment insecurity and experiential avoidance. These intensive interventions make use of attachment processes to increase experiential acceptance and psychological flexibility. Integrated treatment interventions including group dialectical behavior therapy, third-wave cognitive therapy interventions, and mentalization-based approaches are designed to address components of emotion regulation. Moreover, these programmatic interventions take place in a therapeutic milieu that not only accentuates self-regulation of behaviors but also encourages improved social cognition in a culture of greater social acceptance of expressed emotion. Given its intensity, the milieu-based program has the potential to accelerate experiential learning such that a substantial proportion of patients make significant gains over the course of relatively brief treatment.

The mediation analysis revealed that experiential avoidance at admission influenced the relationship between both forms of insecure attachment and improvement in emotion regulation. This finding provides a possible treatment target, namely, improving greater acceptance of distressing emotion in conjunction with greater flexibility and effectiveness in its interpersonal expression. Promoting curiosity and acceptance of internal experiences among individuals with experiential avoidance may create opportunities for reflecting on the interpersonal sources of emotional distress and may lead to opportunities to gain emotion regulation skills. It is assumed that influencing EA through treatment intervention is a more expedient route to improving emotion regulation than attempting to significantly alter attachment style within the context of a 4–8 week treatment. The findings dovetail with a meta-analysis of psychodynamic psychotherapy showing that facilitating patient experience and expression of emotion is associated with improvement [18]. The findings also are consistent with prior research on Acceptance and Commitment Therapy demonstrating that amelioration of experiential avoidance was associated with improvement in patients being treated for social anxiety disorder in individual [86] and group [87] modalities. In extending prior research to an inpatient setting, the present study addresses a growing demand for outcomes research on serious mental illness associated with an increasing awareness of its prevalence, cost, and burden [88, 89]. Concomitantly, this inpatient treatment setting provides an opportunity to explore interpersonal processes that are likely to be evident in a wide range of treatment approaches addressing impairments in emotion regulation.

This study has significant strengths including the sample size, use of validated measures, as well as the treatment duration and intensity, and interpersonal richness of the inpatient treatment program is designed to make use of attachment processes in addressing experiential avoidance and emotion regulation. Yet, the research protocol does not include assessment of specific treatment interventions, such that change cannot be directly tied to interventions. The partial overlap between constructs of emotion regulation and experiential avoidance (specifically the DERS subscale of non-acceptance of emotion regulation and EA) leaves open the question of which interventions targeting EA may have the greatest impact on emotion regulation. In addition, although the mediation analysis yielded clear-cut results, model fit statistics were suboptimal, indicating that other important factors contribute to improved emotion regulation. The effort to identify potential covariates of emotion regulation change was limited to three available variables, none of which appeared to impact rates of change. Specifically, it is plausible that factors such as patient, hospital unit, and therapist characteristics, as well as process and adherence factors are likely exerting an influence on the outcome. This speculation attests to the need for further research with a broader data capture, including post-discharge follow-up to assess the durability of treatment gains.

## Conclusions

The present findings show that impairment in emotional regulation is prominent in a diagnostically heterogeneous group of psychiatric inpatients and that a several-week intensive treatment program is associated with substantially improved emotion regulation. On the premise that the treatment studied includes group-oriented interventions in a therapeutic milieu, theoretical literature suggested that not only self-regulation but also interpersonal regulation would contribute to improved emotion regulation. Accordingly, we included security of attachment as a predictor of change and hypothesized that secure attachment would be associated with experiential acceptance, which we construed as a cornerstone of emotion regulation. We found that both primary forms of insecure attachment, anxiety and avoidance, were associated with experiential avoidance and, moreover, experiential avoidance fully mediated the relation between attachment insecurity and improvement in emotion regulation. These findings imply that helping patients be more aware and expressive of their emotions in close relationships is a potential pathway from insecure attachment to improved emotion-regulation capacities. Intensive, psychotherapeutically oriented inpatient treatment provides an exceptionally rich social setting in which to foster interpersonal regulation of emotion, which we infer accounts for the extent of improvement in a matter of several weeks. Yet these findings likely would generalize to a wider range of residential, day-patient, and intensive-outpatient treatment settings, as well as group psychotherapy more generally. Hence replicating these findings in less intensive treatment settings would be worthwhile.

## Authors’ information

Drs. Allen, Frueh, Ellis & Oldham designed the large-scale study from which the data was drawn. Ms. Szlykh, and Drs. Fowler, Hart, and Allen designed the current study. Dr. Fowler conducted the statistical analyses. Drs. Fowler and Allen wrote the draft. Drs. Frueh Ellis & Oldham added substantial elements to the final draft.

## Abbreviations

BPD: Borderline Personality Disorder
SMI: Severe Mental Illness
DERS: Difficulties in Emotion Regulation Scale
EA: Experiential Avoidance
RQ: Relationship Questionnaire
AAQ-II: Acceptance and Action Questionnaire-II
SCID-I and SCID-II: Structured Clinical Interview for DSM-IV Axis I and Axis II Disorders.
