# Erratum to: intensive inpatient treatment improves emotion-regulation capacities among adults with severe mental illness

**DOI:** 10.1186/s40479-015-0027-8

**Published:** 2015-03-20

**Authors:** J Christopher Fowler, Jon G Allen, John M Hart, Hannah Szlyk, Thomas E Ellis, B Christopher Frueh, John M Oldham

**Affiliations:** The Menninger Clinic, 12301 Main Street, Houston, TX 77035 USA; Baylor College of Medicine, One Baylor Plaza, Houston, TX 77030 USA; Department of Psychology, University of Hawaii, 200 West Kawili St, Hilo, HI 96720 USA

After publication of this research article [1], the authors noticed that the author name Hanna Szlykh should instead be stated as Hannah Szlyk.
